# One-fourth of COVID-19 patients have an impaired pulmonary function after 12 months of disease onset

**DOI:** 10.1371/journal.pone.0290893

**Published:** 2023-09-11

**Authors:** Hugo D. G. van Willigen, Elke Wynberg, Anouk Verveen, Maartje Dijkstra, Bas J. Verkaik, Orlane J. A. Figaroa, Marianne C. de Jong, Annelou L. I. P. van der Veen, Agata Makowska, Nelleke Koedoot, Pythia T. Nieuwkerk, Anders Boyd, Maria Prins, Menno D. de Jong, Godelieve J. de Bree, Joost G. van den Aardweg, the RECoVERED Study Group

**Affiliations:** 1 Department of Medical Microbiology & Infection Prevention, Amsterdam UMC Location University of Amsterdam, Amsterdam, the Netherlands; 2 Amsterdam Institute for Infection and Immunity, Amsterdam, the Netherlands; 3 Department of Infectious Diseases, Public Health Service of Amsterdam, Amsterdam, the Netherlands; 4 Department of Medical Psychology, Amsterdam UMC Location University of Amsterdam, Amsterdam, the Netherlands; 5 Amsterdam Public Health Research Institute, Amsterdam, the Netherlands; 6 Department of Internal Medicine, Amsterdam UMC Location University of Amsterdam, Amsterdam, the Netherlands; 7 Stichting HIV Monitoring, Amsterdam, the Netherlands; 8 Department of Pulmonary Medicine, Amsterdam UMC Location University of Amsterdam, Amsterdam, the Netherlands; Kyung Hee University School of Medicine, REPUBLIC OF KOREA

## Abstract

**Background:**

There is increasing data that show a persistently impaired pulmonary function upon recovery after severe infection. Little is known however about the extent, recovery and determinants of pulmonary impairment across the full spectrum of COVID-19 severity over time.

**Methods:**

In a well characterized, prospective cohort of both hospitalised and non-hospitalised individuals with SARS-CoV-2 infection, the RECoVERED study, pulmonary function (diffusing capacity for carbon monoxide (DLCO)) and spirometry) was measured until one year after disease onset. Additionally, data on sociodemographics, clinical characteristics, symptoms, and health-related quality of life (HRQL) were collected. Pulmonary function and these determinants were modelled over time using mixed-effect linear regression. Determinants of pulmonary function impairment at 12 months after disease onset were identified using logistic regression.

**Findings:**

Between May 2020 and December 2021, 301 of 349 participants underwent at least one pulmonary function test. After one year of follow-up, 25% of the participants had an impaired pulmonary function which translates in 11%, 22%, and 48% of the participants with mild, moderate and severe/critical COVID-19. Improvement in DLCO among the participants continued over the period across one, six and twelve months. Being older, having more than three comorbidities (p<0·001) and initial severe/critical disease (p<0·001) were associated with slower improvement of pulmonary function over time, adjusted for age and sex. HRQL improved over time and at 12 months was comparable to individuals without impaired pulmonary function.

**Interpretation:**

The prevalence of impaired pulmonary function after twelve months of follow-up, was still significant among those with initially moderate or severe/critical COVID-19. Pulmonary function increased over time in most of the severity groups. These data imply that guidelines regarding revalidation after COVID-19 should target individuals with moderate and severe/critical disease severities.

## Introduction

In the past years there have been several coronavirus outbreaks in which pulmonary function and pulmonary sequelae after infection were documented. Patients who recovered from Middle East Respiratory Syndrome (MERS) or Severe Acute Respiratory Syndrome (SARS) pneumonia experienced significant impaired pulmonary function lasting from months to years [1]. As in previous coronavirus outbreaks, also upon SARS-CoV-2 infection, an impaired pulmonary function persists months after disease onset [2–5]. The current literature shows that the most evident pulmonary function abnormality caused by SARS-CoV2 infection is a reduced diffusing capacity for carbon monoxide (DLCO) in association with reduced alveolar volume (VA). The extent of reduced DLCO/VA typically depends on the severity of the illness during the acute phase of infection. Yet this finding is based mainly on studies in hospitalised patients that includes patients admitted to the intensive care unit (ICU). Furthermore, the risk of impaired pulmonary function after SARS-CoV-2 infection is higher for patients with severe disease, prolonged duration of hospital admission, mechanical ventilation, or ICU admission. Few longitudinal studies show that pulmonary function after SARS-CoV-2 infection can improve between eight and twelve months of follow-up [6–8]. Since to a large extent current studies focus on admitted patients, more information is needed about the extent and recovery of pulmonary impairment across the full spectrum of COVID-19. Furthermore, there are concerns about the clinical impact of this impaired pulmonary function after SARS-CoV-2 infection. In this context, reductions in pulmonary function were associated with reduced exercise tolerance in a couple of studies [9–11]. In addition to these studies, a better is insight in the association between pulmonary function over time after infection, quality of life or post COVID-19 symptoms is relevant for further improvement of guidelines regarding post-COVID-19 revalidation.

In the present study we aim to fill this gap by performing a longitudinal analysis of pulmonary function and associated factors in the RECoVERED cohort study [12]. The RECoVERED study is an observational study in which patients with mild, moderate and severe SARS-CoV-2 infection are followed longitudinally and pulmonary function, among a broad spectrum of other variables, is measured at regular intervals. Our previous study in the RECoVERED study shows that a significant proportion of study participants who reported symptoms at disease onset, such as fatigue, dyspnoea, and myalgia, still suffered from these symptoms at one year after disease onset [12]. This raised the question whether persistent symptoms may be associated with an impaired pulmonary function after a SARS-CoV-2 infection. In the present study, we investigated the kinetics of pulmonary function over time and associations between impaired pulmonary function, health-related quality of life (HRQL), symptoms and other associated factors across mild, moderate and severe SARS-CoV-2 disease severities.

## Materials and methods

### Study design

The RECoVERED cohort study is a prospective cohort study that describes the immunological, clinical and psychosocial sequelae of a SARS-CoV-2 infection. Inclusion criteria were an age from 16 to 85 years and a laboratory confirmed SARS-CoV-2 infection. Participants were enrolled in the municipal region of Amsterdam, the Netherlands, from May 2020 until the end of June 2021. The COVID-19 diagnosis was based on positive polymerase chain reaction (PCR). Non-hospitalised participants were identified from notification data at the Public Health Service of Amsterdam (PHSA) and enrolled within seven days of diagnosis. Additionally, participants who were hospitalised were approached on the COVID-19 wards of two academic hospitals in Amsterdam. Between 11 May 2020 and 30 June 2020, a limited number of hospitalised patients were included retrospectively within three months following SARS-CoV-2 diagnosis. The study design, enrolment and follow-up of participants have been described in detail elsewhere (S1 Fig). RECoVERED was approved by the medical ethical review board of the Amsterdam University Medical Centres (NL73759.018.20). All participants provided written informed consent.

In the present study, we included all participants who had at least one pulmonary function measurement during follow-up as of December 2021.

### Study procedures

Data regarding socio-demographic characteristics and past medical history were collected by patient interview and reviewing medical records. A symptom questionnaire on the presence and duration of symptoms was collected through participant interview in the first month of follow-up, based on the World Health Organisation [WHO] Case Report Form [13]. Thereafter, the questionnaire was administered monthly online, to be completed by participants.

Physical signs during the acute phase of the infection, including heart rate (HR), respiratory rate (RR) and oxygen saturation (SpO_2_), were measured or retrieved from hospital records at enrolment (D0), seven days (D7) and one month (M1) after enrolment.

### Pulmonary function testing

Pulmonary function was measured according to the American Thoracic Society (ATS)–European Respiratory Society (ERS) guidelines [14]. At 28 days, six months, and twelve months after enrolment participants underwent standard pulmonary function testing (PFT). The following lung function parameters were measured: forced vital capacity (FVC), forced expiratory volume in one second (FEV_1_), vital capacity (VC), and alveolar volume (VA). In addition, pulmonary diffusion capacity for carbon dioxide (DLCO) was measured as an absolute value. Before each PFT, haemoglobin was measured and used in the measurement of DLCO. Reference values from the Global Lung Function Initiative (GLI) Equations and ERS were used for the spirometric and diffusion capacity parameters [15]. FVC, FEV_1_, VC, VA and DLCO were presented as percentage of predicted, according to guidelines these values were based on sex, age and height [15, 16]. The predicted DLCO/VA (KCO) was also included for the calculation of the predicted DLCO. Alveolar volume was derived from dilution of methane during the manoeuvre. Impaired pulmonary function was defined as a percentage of predicted taken to be equal to the 5th percentile of a healthy, non-smoking population. These PFTs were conducted at the Amsterdam UMC (location AMC) using CareFusion MasterScreen^®^ PFT with SentrySuite software (Vyaire, Wuerzburg, Germany).

On the day of PFT the spirometer was calibrated, including registration of barometric pressure and temperature. During the PFT trained members of the study staff coached the participant, while routinely being supervised by a pulmonary technician. Pulmonologists within the study group were responsible for test validation and interpretation. In the analysis, the highest value of the three attempts was used and differences to the PFT predicted values and lower limit of normal values were calculated. To ensure measurement quality, the maximum inspiration during spirometry and during the single-breath CO technique were not allowed to differ by more than 5%. Lung function tests performed under similar methods before COVID-19 diagnosis were gathered, if available.

### Health-related quality of life (HRQL)

Participants were asked to complete the Medical Outcomes Studies Short Form 36-item Health Survey (SF-36), a self-administered questionnaire containing 36 items to assess health-related quality of life, at one and twelve months of follow-up [17]. The questionnaire measures health on eight multi-item dimensions, covering functional status, well-being, and overall evaluation. Participants were asked to rate their responses on three- or six-point scales in six of the eight dimensions. Item scores were coded, summed, and transformed for each dimension, resulting in a scale ranging from 0 (i.e., worst health) to 100 (i.e., best health).

### Definitions

Based on the WHO COVID-19 disease severity criteria, clinical severity groups were defined as: mild disease, having an RR <20/min, and SpO2>94% on room air on D0, D7, and/or M1; moderate disease, having an RR 20-30/min or SpO2 90–94%, or receiving oxygen therapy at D0, D7, or M1; severe disease, having an RR>30/min or SpO2<90% at D0, D7, or M1; critical disease, ICU admission due to COVID-19 at any time point. Disease onset was defined as the first day of symptom onset in symptomatic patients or the day of diagnosis in asymptomatic patients. BMI (kg/m^2^) was categorised as: <25, underweight or normal weight; 25–30, overweight; >30, obese. Symptoms reported in the first month after overall disease onset were defined as acute symptoms due to COVID-19. The predicted lung function was regarded as impaired if a measured pulmonary value was below the lower limit of normal (LLN), which was already based according to pre-defined factors (e.g. sex, age, height) [14–16].

### Statistical analysis

Clinical and sociodemographic characteristics were compared between moderate/severe and mild disease severity groups. Continuous variables were given as medians and interquartile ranges, compared using the Mann-Whitney test. Categorical variables were given as frequencies and percentages, compared using the Pearson χ^2^ or Fisher exact test, when appropriate [18].

Lung function was modelled overtime using mixed-effects linear regression [19]. The kinetics (per 6 months) of lung function was obtained by including a fixed-effect for time, while adding a random intercept to account for between-patient variation at enrolment [20, 21]. In multivariable analysis, we produced increasingly complex multivariable models where variables were grouped as follows: model 1, clinical and sociodemographic characteristics; Model 2, addition of acute phase COVID-19 symptoms (as listed in Table 1); and model 3, addition of COVID-19 clinical severity. At each model, variables were selected using a backwards stepwise approach in which variables with a p-value <0·20 from a Wald χ^2^ test were retained. Age, sex, and time since disease onset were forced in all models.

**Table 1:** Socio-demographic, clinical (baseline and COVID-19-related) and study characteristics of RECoVERED study participants who conducted a pulmonary function test between May 2020-December 2021, in Amsterdam, the Netherlands.

	Total	Mild	Moderate	Severe/Critical	p-value
	N = 301	N = 89	N = 133	N = 79	
Sex					0.32
Male	170 (56%)	45 (51%)	76 (57%)	49 (62%)	
Female	131 (44%)	44 (49%)	57 (43%)	30 (38%)	
Age, years	51 (36–62)	40.0 (27.0–53.0)	49.0 (34.0–61.0)	60.0 (50.0–66.0)	<0.001
BMI, kg/m2^†^	25.8 (23.2–29.4)	24.4 (22.8–27.3)	25.7 (23.2–29.4)	27.5 (25.1–33.3)	<0.001
BMI category					<0.001
Normal weight	126 (42%)	52 (58%)	55 (41%)	19 (24%)	
Overweight	102 (34%)	23 (26%)	46 (35%)	33 (42%)	
Obese	68 (23%)	13 (15%)	30 (23%)	25 (32%)	
Missing	5 (2%)	1 (1%)	2 (2%)	2 (3%)	
Smoking					0.12
Non-smoker	187 (62%)	55 (62%)	76 (57%)	56 (71%)	
Smoker	20 (7%)	8 (9%)	11 (8%)	1 (1%)	
Ex-smoker	88 (29%)	23 (26%)	44 (33%)	21 (27%)	
Missing	6 (2%)	3 (3%)	2 (2%)	1 (1%)	
Number of comorbidities*					<0.001
0	140 (47%)	56 (63%)	63 (47%)	21 (27%)	
1	87 (29%)	23 (26%)	34 (26%)	30 (38%)	
2	31 (10%)	5 (6%)	15 (11%)	11 (14%)	
3 or more	43 (14%)	5 (6%)	21 (16%)	17 (22%)	
Cardiovascular disease	77 (26%)	11 (12%)	32 (24%)	34 (44%)	<0.001
Diabetes	34 (11%)	4 (4%)	10 (8%)	20 (26%)	<0.001
Asthma and/or COPD	34 (11%)	8 (9%)	17 (13%)	9 (12%)	0.68
Other chronic pulmonary disease	12 (4%)	1 (1%)	4 (3%)	7 (10%)	0.020
Cancer	18 (6%)	6 (7%)	8 (6%)	4 (5%)	0.91
Other comorbidities	67 (22%)	12 (14%)	35 (27%)	20 (26%)	0.060
**Clinical features of SARS-CoV-2 infection**					
Symptom status upon disease onset					0.45
Symptomatic	299 (99%)	88 (99%)	133 (100%)	78 (99%)	
Asymptomatic **	2 (1%)	1 (1%)	0 (0%)	1 (1%)	
Hospital admission	141 (47%)	4 (4%)	61 (46%)	76 (96%)	<0.001
Duration of hospital admission	6 (4–10)	3 (2–6)	5 (4–8)	9 (4–14)	<0.001
ICU admission	39 (13%)	0 (0%)	0 (0%)	39 (49%)	<0.001
Duration of ICU admission^#^	6 (4–13)			6 (4–13)	
Received suppl. oxygen therapy	86 (29%)	1 (0%)	56 (42%)	29 (37%)	<0.001
Received MV (non-invasive)	15 (5%)	0 (0%)	0 (0%)	15 (19%)	
Received MV (invasive)	29 (10%)	0 (0%)	0 (0%)	29 (37%)	
Received Dexamethason	70 (25%)	0 (0%)	34 (27%)	36 (46%)	<0.001
Received Tocilluzimab	14 (10%)	0 (0%)	2 (3%)	12 (16%)	0.022
Days from disease onset to COVID-19 diagnosis	4 (2–9)	3 (1–8)	4 (2–9)	7 (2–10)	0.26
Time from start disease to hospitalisation	9 (7–13)	49 (16–85)	9 (8–14)	9 (7–12)	0.11
Time from start disease to ICU admission^#^	10 (7–12)			10 (7–12)	
Time from hospitalisation to ICU admission^#^	1 (0–3)			1 (0–3)	
Acute presence (<28 days from disease onset) of symptom:					
Dyspnoea	138 (61%)	31 (39%)	76 (72%)	31 (78%)	<0.001
Cough	167 (75%)	49 (61%)	85 (81%)	33 (87%)	0.002
Fever	140 (64%)	42 (53%)	73 (70%)	25 (68%)	0.052
Myalgia	143 (64%)	53 (66%)	71 (68%)	19 (49%)	0.095
Wheezing	59 (26%)	8 (10%)	31 (29%)	20 (53%)	<0.001
Fatigue	193 (87%)	68 (84%)	92 (88%)	33 (92%)	0.50

BMI, Body mass index; ICU, Intensive Care Unit; MV, mechanical ventilation.

^†^ Normal BMI group includes 3 individuals with BMI between 18.0 and 18.5 kg/m2.

^#^ Only those admitted to the Intensive Care Unit

* COVID-related comorbidities are based on WHO Clinical Management Guidelines [13] and include: cardiovascular disease (including hypertension), chronic pulmonary disease (excluding asthma), renal disease, liver disease, cancer, immunosuppression (excluding HIV, including previous organ transplantation), previous psychiatric disease and dementia.

** Asymptomatic participants were diagnosed with a positive PCR-test during routine testing, but did not had any COVID-19 related symptoms at the time of testing.

Impaired pulmonary function that persisted after 12 months of disease onset was modelled as an outcome using logistic regression. The same procedure as described above was used to construct the multivariable models for these analyses. Adjusted odds ratios (ORs) and their 95% CIs are presented.

In post-hoc analysis, we determined whether severe lung impairment affected HRQL over time. We modelled continuous HRQL using mixed-effects linear regression. We included time points (i.e., month one and month twelve), lung function severity (i.e., severe or not severe) and the interaction between the two as fixed covariates, while adding a random intercept across patients. This model was adjusted for age and sex. We tested the difference between severity groups at each time point using Wald χ^2^ test. *P*-values were two-sided and a *P*-value of <0·05 was considered statistically significant. Data were analysed using Stata statistical software (v15.1, College Station, TX, USA).

## Results

### Characteristics of study participants

In total, 349 participants were enrolled in the RECoVERED cohort study. By December 2021, 301 (86%) participants underwent at least one PFT during follow-up and were included in the present study. Participant flow is described in S2 Fig. Of the 301 participants, 89 (30%) experienced mild, 133 (44%) moderate, and 79 (26%) severe/critical disease severity. The median age was 51 year (IQR, 36–62 year) and 170 (56%) were men (Table 1). Older age (p<0·001), higher BMI (p<0·001), number of comorbidities in general (p<0·001), cardiovascular disease (CVD) (p<0·001), diabetes (DM) (p<0·001), and chronic pulmonary diseases other than asthma or COPD (p = 0·02) were more prevalent in the severe/critical compared to mild severity group. Of the 301 participants included in the present analysis, 141 (47%) were hospitalized. On admission, 86 (29%) of the hospitalized participants received low flow oxygen therapy, 15 required non-invasive (high flow) ventilation and 29 required invasive mechanical ventilation. A total of 39 participants (13%) were admitted to the ICU, with a median stay of 6 (IQR 4–13) days. Overall, 70 participants (23%) received dexamethasone and 14 participants (10%) received Tociluzimab.

### Longitudinal dynamics of pulmonary function

**Fig 1:**
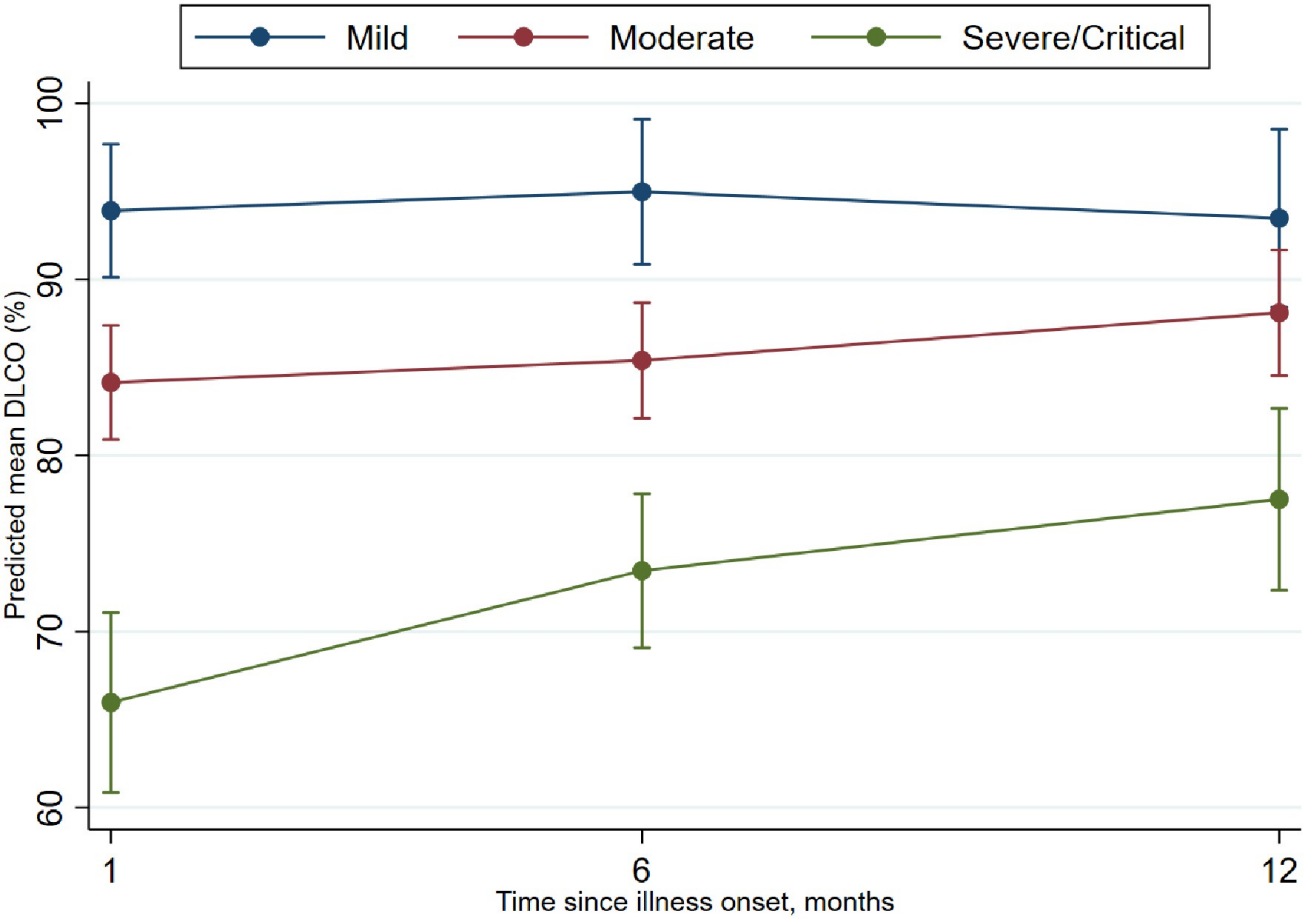
Longitudinal change in diffusion capacity for participants with mild (blue), moderate (red) and severe (green) disease after disease onset. The y-axis shows median DLCO corrected for haemoglobin and the x-axis is time in months after disease onset. Vertical bars represent 95% confidence intervals.

First, we set out to determine the dynamics in pulmonary function over time in the first year post disease onset (Table 2 and S1 Table). The time points included in these analyses were 1, 6 and 12 months after disease onset. Significant differences across the three severity groups (mild, moderate, severe) in spirometry values (FEV1, FVC and VC) were apparent only at month 1. However, the diffusion capacity differences between groups were observed beyond month 1. At the first month, a DLCO below the lower limit of normal (LLN, see methods section) was observed in six (26%) participants with mild, 21 (23%) with moderate and 25 (74%) with severe/critical disease severity (Table 2). In the mild disease group, no statistically significant improvement in diffusion capacity occurred up to 1 year after disease onset. Among participants with moderate disease, improvement of spirometry was observed between one and six months, but no further improvement occurred between six months and 1 year. Participants with moderate and severe diseases had an improvement in DLCO which continued over the entire year until 12 months post disease onset (Fig 1).

**Table 2:** Pulmonary function for each time point (1, 6 and 12 months after disease onset) for participants stratified in the different disease severity groups (mild, moderate and severe/critical).

Spirometry					
	Total	Mild	Moderate	Severe/critical	p-value*
	N = 301	N = 89	N = 133	N = 79	
FVC month 1 below LLN	30/213 (14%)	3/74 (4%)	12/101 (12%)	15/38 (39%)	<0.001
FVC month 6 below LLN	20/224 (9%)	2/70 (3%)	12/104 (12%)	6/50 (12%)	0.077
FVC month 12 below LLN	11/128 (9%)	2/33 (6%)	4/66 (6%)	5/29 (17%)	0.23
FEV_1_ month 1 below LLN	28/213 (13%)	3/74 (4%)	13/101 (13%)	12/38 (32%)	<0.001
FEV_1_ month 6 below LLN	27/224 (12%)	5/70 (7%)	12/104 (12%)	10/50 (20%)	0.12
FEV_1_ month 12 below LLN	20/128 (16%)	3/33 (9%)	10/66 (15%)	7/29 (24%)	0.26
VC month 1 below LLN	26/213 (12%)	3/74 (4%)	8/101 (8%)	15/38 (39%)	<0.001
VC month 6 below LLN	18/224 (8%)	2/70 (3%)	10/104 (10%)	6/50 (12%)	0.12
VC month 12 below LLN	10/128 (8%)	2/33 (6%)	3/66 (5%)	5/29 (17%)	0.14
**Single-breath carbon monoxide uptake in the lung / diffusion capacity**					
DLCO month 1 below LLN	52/197 (26%)	6/70 (9%)	21/93 (23%)	25/34 (74%)	<0.001
DLCO month 6 below LLN	51/195 (26%)	4/54 (7%)	22/90 (24%)	25/51 (49%)	<0.001
DLCO month 12 below LLN	31/122 (25%)	3/28 (11%)	14/65 (22%)	14/29 (48%)	0.004
VA month 1 below LLN	61/199 (31%)	10/71 (14%)	28/94 (30%)	23/34 (68%)	<0.001
VA month 6 below LLN	3/195 (2%)	0/55 (0%)	2/90 (2%)	1/50 (2%)	0.62
VA month 12 below LLN	23/104 (22%)	2/24 (8%)	14/54 (26%)	7/26 (27%)	0.18

*Differences between groups for each pulmonary function parameter were tested and p<0.05 were considered significant.

FVC, Forced vital capacity; FEV_1_, forced expiratory volume in 1 second; VC, vital capacity; DLCO, corrected pulmonary diffusion capacity; VA, alveolar volume; LLN, lower limit of normal.

Next, we determined to what extent factors such as clinical and sociodemographic characteristics (model 1), acute phase COVID-19 symptoms (added to model 1 = model 2) and COVID-19 clinical severity (added to model 2 = model 3) were associated with DLCO. In the first linear mixed-effects regression model, age (β -0·40, 95%CI: -0·54, -0·25) p<0·001), female sex (β -3·94, 95%CI: -7·23, -0·66) p = 0·019), and higher number of comorbidities (β -6·49, 95%CI: -8·84- -4·15) p<0·001) were associated with rate of improvement of DLCO over time. In model 2, age and higher number of comorbidities remained significant when adjusting for acute phase clinical symptoms. While rhinitis, dyspnoea and cough in the acute phase was significantly associated with DLCO over time in univariable analysis, these symptoms did not remain statistically significantly associated with the outcome in multivariable model 2. When including clinical severity in model 3, age (β -0·44, 95%CI: -0·60- -0·27) p<0·001), female sex (β -5·93, 95%CI: -10·30- -1·56) p = 0·01), higher number of comorbidities (β -5·42, 95%CI: -8·22- -2·62) p<0·001), and initial disease severity (β -6·42, 95%CI: -9·77- -3·08) p<0·001) were significantly associated with a slower improvement of DLCO over time (S2 Table).

### Factors associated with impaired pulmonary function at 12 months after disease onset

Given the markedly reduced DLCO we observed at 12 months after disease onset (11%, 22%, and 48% for mild, moderate, and severe/critical disease severity, respectively, Table 2) we analysed factors associated with this reduced DLCO (S3 Table). When adjusted for age and sex in multivariable analysis, risk factors associated with a DLCO below LLN at 12 months were severe COVID-19 (OR = 2·62, 95%CI: 1·19–5·82) and the presence of any comorbidity (OR = 3·23, 95%CI: 1·78–5·85).

### Association between pulmonary function and HRQL

HRQL was measured at 1 and 12 months after disease onset and consist of measures of health on eight multi-item dimensions, covering functional status, well-being, and overall evaluation (Table 3). When adjusting for age and sex, participants with a DLCO below LLN in the first month after disease onset scored significantly lower on the physical functioning (β -20·27, 95%CI: -27·91- -12·63) and general health (β -10·34, 95%CI: -17·21- -3·47) dimensions of the SF-36. Also FVC below LLN at month 1, was associated with lower physical functioning (β -39·38, 95%CI: -48·87- -29·89) and general health (β -15·34, 95%CI: -24·04- -6·64). In addition, FEV_1_ below LLN was associated with a lower physical functioning dimension score (β -20·75, 95%CI: -30·76- -10·73). In individuals with impaired pulmonary function (spirometry as well as DLCO) at 12 months after disease onset, HRQL on all dimensions was not different compared to participants without impaired pulmonary function at 12 months.

**Table 3:** Health-related quality of life per time point of different severity groups after disease onset.

Health-related quality of life item scores					
	Total	Mild	Moderate	Severe/critical	p-value
	N = 301	N = 89	N = 133	N = 79	
Physical Functioning month 1	80 (50–95)	95 (80–100)	73 (45–95)	55 (30–70)	<0.001
Physical Functioning month 12	95 (75–100)	100 (93–100)	90 (70–100)	80 (65–100)	0.004
Role Functioning-Physical month 1	25 (0–100)	50 (0–100)	25 (0–50)	0 (0–75)	0.002
Role Functioning-Physical month 12	100 (75–100)	100 (100–100)	100 (75–100)	100 (25–100)	0.14
Bodily Pain month 1	72 (41–100)	84 (62–100)	62 (41–80)	62 (41–100)	<0.001
Bodily Pain month 12	84 (62–100)	100 (84–100)	100 (72–100)	74 (51–100)	0.007
Social Functioning month 1	63 (38–88)	75 (50–100)	50 (38–63)	50 (38–88)	<0.001
Social Functioning month 12	88 (75–100)	100 (88–100)	88 (63–100)	88 (63–100)	0.027
Mental Health month 1	76 (60–88)	78 (62–88)	72 (56–84)	80 (68–92)	0.016
Mental Health month 12	84 (72–92)	88 (80–96)	76 (64–92)	84 (76–96)	0.053
Role Functioning-Emotional month 1	100 (33–100)	100 (33–100)	67 (0–100)	67 (0–100)	0.11
Role Functioning-Emotional month 12	100 (67–100)	100 (100–100)	100 (67–100)	100 (67–100)	0.051
Vitality month 1	50 (35–65)	60 (45–78)	43 (30–60)	40 (35–55)	<0.001
Vitality month 12	75 (55–85)	80 (70–85)	65 (55–80)	70 (55–80)	0.008
General Health Perceptions month 1	65 (50–80)	80 (65–90)	63 (50–75)	55 (35–70)	<0.001
General Health Perceptions month 12	70 (50–85)	80 (63–90)	65 (45–80)	65 (45–80)	0.014

## Discussion

This study aimed to investigate and evaluate the kinetics of pulmonary function over time in a cohort of patients with a varying degree of SARS-CoV-2 severity. Our findings indicate that one-fourth of patients still have impaired the single-breath diffusing capacity 12 months after infection. Nevertheless, significant increases in DLCO, VA, and FVC were observed among those with an initially moderate or severe/critical COVID-19, suggesting that COVID-19-associated lung damage can be reversed to a certain degree. Reassuringly, these individuals were also able to achieve a level of HRQL that was not different from those without impaired pulmonary function.

The impaired pulmonary function observed in our study was mainly driven by an abnormal diffusion capacity and persisted up to 12 months of disease onset. Abnormalities in DLCO were mostly found in patients in the moderate and severe disease severity groups, which was in accordance with other studies [6–8, 10]. These findings are more suggestive of restrictive lung function origin, as opposed to volume-adjusted diffusion capacity. The association between severity of COVID-19 and restrictive pulmonary function has also been observed in previous studies with follow-up of up to one year [8, 22]. Over 12 months of follow-up, an ongoing improvement in pulmonary function was observed in the severe/critical disease severity group. In the moderate disease severity group this improvement in pulmonary function began to wane after the first 12 months for individuals. This waning is likely the result of less involvement of the lower respiratory tract and thus less damage of the lung parenchyma during the acute phase of the disease in mild and moderate disease severities. Zhang et al. observed a comparable improved in pulmonary function in hospitalized patients up to 1 year after disease onset [23].

Pulmonary fibrosis has been found to persist months and even years after COVID-19 infection [24]. The persisting restrictive pulmonary function that we found in a significant proportion of participants after severe infection, could be associated with persisting radiological abnormalities that are compatible with pulmonary fibrosis. Because in our study we did not routinely perform a CT scan in the months after recovery from acute disease, we cannot specify the proportion of participants with pulmonary fibrosis.

One month after disease onset, study participants with impaired pulmonary function scored lower on physical functioning and general health. In part this may be due to muscle weakness developed during ICU admission, which has been associated with substantial impairments in physical function, restrictive pulmonary function, and quality of life [25]. In addition, at the end of the follow-up the patient group with impaired pulmonary function had an increase in HRQL. When compared with the HRQL of individuals without impaired pulmonary function, there was no significant difference. This observation can be a result of rehabilitation of patients with impaired pulmonary function [26]. However, the increase in HRQL over time seems not relate to all patients who were experiencing persistent symptoms one year after disease onset [27].

This study has several limitations. Retrospective enrolment of ICU patients obviously meant that only individuals who survived could be included, which might have introduced survival bias. In addition, patients who were in a life-threatening situation were unlikely to have been enrolled in the study. Hence, our findings might not be generalizable to the larger group of COVID-19 patients with severe/critical disease severity. Secondly, newly developed or integrated treatments became available (e.g. the use of dexamethasone and Tocilizumab) during the pandemic. Furthermore, a small number (n = 34) of study participants received newly prescribed corticosteroids over time after the initial COVID-19 illness episode (indication and dosage was not further defined). These data raise the possibility that accelerated recovery from COVID-19 disease could have occurred over the course of the study. As a result, impairment of pulmonary function might have been less severe than it would have if the infection had run its natural course. Nevertheless, our results clearly demonstrate that impaired lung function is a common problem after 12 months of infection. Due to the lack of pre-COVID-19 PFT measurements, we were unable to assess what proportion of impaired lung function was directly attributable to SARS-CoV-2 infection as compared to pre-existing pulmonary comorbidities. Lastly, GLI reference equations and values are limited to Caucasians. This may lead to an increase in between-individual variability, thereby underestimating the LLN, but the predicted value should not be affected [16]. Finally, the study is performed in a Western European country and data may not be directly translated to patients with COVID-19 from other geographical or ethnic background.

## Conclusions

In conclusion, single-breath diffusing capacity improvement after SARS-CoV-2 infection varied noticeably between the disease severities, while prolonged recovery of pulmonary function was observed in patients who had severe or critical COVID-19. These results showed the extent and recovery of impaired pulmonary functions over time across the full spectrum of disease severity. The found trajectories allows clinicians to benchmark the progression of their patients based particularly on the initial severity of their disease. Guidelines regarding revalidation after COVID-19 should target the first 6 months after disease onset and in particular individuals with moderate and severe COVID-19. Secondly, the severity, duration of impaired pulmonary function and association with other COVID-19 sequelae illustrates the impact and burden lived by individuals after COVID-19. In addition, longer follow-up is needed to investigate the extent of recovery in individuals with severe/critical COVID-19.

## Supporting information

S1 Table Pulmonary function presented in z-scores for each time point (1, 6 and 12 months after disease onset) for participants stratified in the different disease severity groups (mild, moderate and severe/critical). (DOCX)

S2 Table Selection of determinants for inclusion in linear mixed models, by model type. (DOCX)

S3 Table Determinants of impaired pulmonary function at 12 months after disease onset. (DOCX)

S1 Fig Overview of data collection in the RECoVERED Study, Amsterdam, the Netherlands. (TIF)

S2 Fig Flow diagram participants who underwent at least one pulmonary function test. (TIF)

S1 Checklist STROBE statement—Checklist of items that should be included in reports of *cohort studies*. (DOC)
