# Supplementary material for: One-fourth of COVID-19 patients have an impaired pulmonary function after 12 months of disease onset
Source: PLoS One. 2023 Sep 11;18(9):e0290893. doi: 10.1371/journal.pone.0290893 (PMC10495003; doi:10.1371/journal.pone.0290893)
Supplement: S1 Table — (DOCX) [file pone.0290893.s001.docx]

| **Spirometry** | | | | | |
| --- | --- | --- | --- | --- | --- |
|  | **Total** | **Mild** | **Moderate** | **Severe/critical** | **p-value^*^** |
|  | **N=301** | **N=89** | **N=133** | **N=79** |  |
| FVC month 1 | 0.07 (-1.04-0.81) | 0.41 (-0.12-1.01) | -0.06 (-1.03-0.79) | -1.16 (-2.11--0.24) | <0.001 |
| FVC month 6 | 0.10 (-0.76-0.80) | 0.40 (-0.00-1.11) | 0.00 (-0.83-0.74) | -0.15 (-1.18-0.41) | 0.001 |
| FVC month 12 | 0.16 (-0.62-0.89) | 0.42 (0.00-1.22) | -0.08 (-0.85-0.90) | -0.07 (-0.88-0.58) | 0.032 |
| FEV_1_ month 1 | -0.06 (-1.10-0.70) | 0.34 (-0.18-0.87) | -0.23 (-1.05-0.63) | -1.08 (-2.02--0.11) | <0.001 |
| FEV_1_ month 6 | -0.00 (-0.88-0.60) | 0.29 (-0.54-0.70) | -0.16 (-0.96-0.63) | -0.14 (-1.53-0.46) | 0.042 |
| FEV_1_ month 12 | -0.15 (-1.06-0.65) | 0.16 (-0.82-0.66) | -0.27 (-0.97-0.66) | -0.20 (-1.58-0.47) | 0.29 |
| VC month 1 | 0.06 (-1.07-0.81) | 0.46 (-0.12-1.02) | -0.16 (-1.05-0.79) | -1.16 (-2.11--0.24) | <0.001 |
| VC month 6 | 0.22 (-0.61-0.86) | 0.44 (-0.00-1.10) | 0.08 (-0.64-0.86) | -0.07 (-1.16-0.57) | 0.003 |
| VC month 12 | 0.29 (-0.53-0.98) | 0.42 (0.00-1.22) | 0.08 (-0.75-0.94) | 0.35 (-0.78-0.74) | 0.16 |
| **Single-breath carbon monoxide uptake in the lung / diffusion capacity** | | | | | |
| DLCO month 1 | -0.91 (-1.75-0.03) | -0.28 (-1.10-0.43) | -0.95 (-1.50--0.33) | -2.48 (-3.06--1.63) | <0.001 |
| DLCO month 6 | -0.94 (-1.70--0.28) | -0.65 (-1.14-0.31) | -0.90 (-1.64--0.25) | -1.64 (-2.49--0.93) | <0.001 |
| DLCO month 12 | -0.74 (-1.64--0.02) | -0.71 (-1.26-0.24) | -0.45 (-1.50-0.12) | -1.45 (-2.44--0.89) | <0.001 |
| VA month 1 | -0.77 (-1.77-0.08) | -0.48 (-0.97-0.17) | -0.81 (-1.76-0.09) | -2.30 (-3.02--0.93) | <0.001 |
| VA month 6 | -0.95 (-1.83--0.18) | -0.52 (-1.02-0.00) | -0.95 (-1.68--0.08) | -1.61 (-2.45--0.88) | <0.001 |
| VA month 12 | -0.74 (-1.47--0.08) | -0.38 (-0.95-0.00) | -0.76 (-1.67--0.18) | -1.22 (-1.63--0.53) | 0.033 |

## S1 Table. Pulmonary function presented in z-scores for each time point (1, 6 and 12 months after disease onset) for participants stratified in the different disease severity groups (mild, moderate and severe/critical)

^*^Differences between groups for each pulmonary function parameter were tested and p<0.05 were considered significant.

FVC, Forced vital capacity; FEV_1_, forced expiratory volume in 1 second; VC, vital capacity; DLCO, corrected pulmonary diffusion capacity; VA, alveolar volume; LLN, lower limit of normal.
