# Supplementary material for: One-fourth of COVID-19 patients have an impaired pulmonary function after 12 months of disease onset
Source: PLoS One. 2023 Sep 11;18(9):e0290893. doi: 10.1371/journal.pone.0290893 (PMC10495003; doi:10.1371/journal.pone.0290893)
Supplement: S2 Table — (DOCX) [file pone.0290893.s002.docx]

## **S2 Table. Selection of determinants for inclusion in linear mixed models, by model type.**

|  | **Determinant** | **Univariable analysis** | | **Multivariable Model 1** | | **Multivariable Model 2** | | **Multivariable Model 3** | |
| --- | --- | --- | --- | --- | --- | --- | --- | --- | --- |
|  |  | **β coef. (95% CI)** | **p-value** | **β coef. (95% CI)** | **p-value** | **β coef. (95% CI)** | **p-value** | **β coef. (95% CI)** | **p-value** |
| **Socio-demographic and medical characteristics at illness onset** | Age | *-0.61 (-0.74--0.48)* | *<0.001* | *-0.40 (-0.54--0.25)* | *<0.001* | *-0.49 (-0.66--0.31)* | *<0.001* | *-0.44 (-0.60--0.27)* | *<0.001* |
|  | Sex |  | 0.72 |  | 0.13 |  | *0.01* |  | *0.01* |
|  | Male | Ref. |  | Ref. |  | *Ref.* |  | *Ref.* |  |
|  | Female | 0.86 (-3.85-5.58) |  | -3.51 (-7.46-0.43) |  | *-5.74 (-10.32--1.16)* |  | *-5.93 (-10.30--1.56)* |  |
|  | BMI group |  | 0.30 |  | NA |  | NA |  | NA |
|  | Normal weight | Ref. |  | NA |  | NA |  | NA |  |
|  | Overweight | -4.86 (-10.09-0.37) |  | NA |  | NA |  | NA |  |
|  | Obese | -2.23 (-8.13-3.67) |  | NA |  | NA |  | NA |  |
|  | Number of high-risk COVID-19 comorbidities at illness onset* |  | *<0.001* |  | *<0.001* |  | *0.001* |  | *<0.001* |
|  | 0 | *Ref.* |  | *Ref.* |  | *Ref.* |  | *Ref.* |  |
|  | 1 | *-11.28 (-16.25--6.32)* |  | -3.85 (-8.78-1.08) |  | 1.33 (-4.51-7.18) |  | -0.04 (-5.51-5.42) |  |
|  | 2 | *-13.59 (-20.06--7.11)* |  | -4.16 (-10.84-2.52) |  | -3.92 (-11.74-3.89) |  | -3.23 (-10.74-4.28) |  |
|  | 3+ | *-34.88 (-42.71--27.05)* |  | *-26.62 (-34.45--18.79)* |  | *-26.89 (-37.83--15.94)* |  | *-27.23 (-37.30--17.17)* |  |
|  | Presence of asthma & COPD | *6.48 (-0.85-13.82)* | *0.08* | NA | NA | NA | NA | NA | NA |
|  | Presence of other pulmonary comorbidities | *-20.30 (-32.25--8.35)* | *0.001* | -7.98 (-18.68-2.72) | 0.14 | NA | NA | NA | NA |
|  | Smoking |  | 0.07 | NA | NA | NA | NA | NA | NA |
|  | Non-smoker | Ref. |  | NA | NA | NA | NA | NA | NA |
|  | Ex-smoker | -4.93 (-10.07-0.21) |  | NA | NA | NA | NA | NA | NA |
|  | Current smoker | 0.03 (-9.35-9.41) |  | NA | NA | NA | NA | NA | NA |
|  | Presence of fibrosis on CT-scan during follow-up | -8.77 (-18.46--0.91) | 0.08 | NA | NA | NA | NA | NA | NA |
| **Clinical severity** | Clinical severity*** |  | *<0.001* |  |  |  |  |  | *<0.001* |
|  | Mild | *Ref.* |  | NA | NA | NA | NA | *Ref.* |  |
|  | Moderate | *-9.24 (-14.20--4.29)* |  | NA | NA | NA | NA | *-5.96 (-10.77--1.15)* |  |
|  | Severe/critical | *-22.85 (-28.54--17.15)* |  | NA | NA | NA | NA | *-13.11 (-20.10--6.12)* |  |

**Continues on the next page.**

|  | **Determinant (selected *a priori*)** | **Univariable analysis** | | **Multivariable Model 1** | | **Multivariable Model 2** | | **Multivariable Model 3** | |
| --- | --- | --- | --- | --- | --- | --- | --- | --- | --- |
|  |  | **β coef (95% CI)** | **p-value** | **β coef . (95% CI)** | **p-value** | **β coef. (95% CI)** | **p-value** | **β coef. (95% CI)** | **p-value** |
| **Clinical features of COVID-19** | Presence of acute fatigue | *-2.64 (-10.57-5.30)* | *0.52* | NA | NA | NA | NA | NA | NA |
|  | Presence of acute rhinitis | *5.73 (0.22-11.24)* | *0.04* | NA | NA | 2.53 (-2.15-7.20) | 0.29 | NA | NA |
|  | Presence of acute wheezing | *-10.64 (-16.57--4.70)* | *<0.001* | NA | NA | *-7.36 (-12.77--1.94)* | *<0.01* | -4.74 (-9.91-0.43) | 0.07 |
|  | Presence of acute dyspnoea | *-9.19 (-14.46--3.93)* | *0.001* | NA | NA | -2.27 (-7.21-2.67) | 0.37 | NA | NA |
|  | Presence of acute cough | *-9.15 (-15.08--3.22)* | *<0.01* | NA | NA | -1.02 (-6.49-4.46) | 0.72 | NA | NA |
|  | Presence of acute fever | *-5.40 (-10.88--0.7)* | *0.05* | NA | NA | NA | NA | NA | NA |
|  |  |  | | | | | | | |

In multivariable analysis, the following variables were removed because they were above the p-value threshold: Model 1: NA; Model 2: Presence of other pulmonary comorbidities (p=0.14), Presence of acute rhinitis (p=0.29), dyspnoa (p=0.37) and cough (p=0.72); Model 3: Presence of acute wheezing (p=0.07).

NA = Not applicable. BMI=Body mass index; OECD= Organisation for Economic Co-operation and Development; LMIC = low- or middle-income country.

* COVID-related comorbidities are based on WHO Clinical Management Guidelines [1] and include: cardiovascular disease (including hypertension), chronic pulmonary disease (excluding asthma), renal disease, liver disease, cancer, immunosuppression (excluding HIV, including previous organ transplantation), previous psychiatric illness and dementia.

** Migration background based on country of birth of participant and that of their parents.

***Clinical severity groups defined as: mild as having a RR<20/min and SpO2 on room air >94% at both day 0 and 7; moderate disease as having a RR 20-30/min, SpO2 90-94% and/or receiving oxygen therapy at day 0 or 7; severe disease as having a RR>30/min or SpO2 < 90% at day 0 or 7; critical disease as requiring ICU admission.

**References**

1. World Health Organization. Clinical management of COVID-19: interim guidance, 27 May 2020. World Health Organization; 2020.
