# Supplementary material for: One-fourth of COVID-19 patients have an impaired pulmonary function after 12 months of disease onset
Source: PLoS One. 2023 Sep 11;18(9):e0290893. doi: 10.1371/journal.pone.0290893 (PMC10495003; doi:10.1371/journal.pone.0290893)
Supplement: S3 Table — (DOCX) [file pone.0290893.s003.docx]

## **S3 Table. Determinants of impaired pulmonary function at 12 months after disease onset.**

|  | **Determinant** | **Univariable analysis** | | **Multivariable analysis** | |
| --- | --- | --- | --- | --- | --- |
|  |  | **OR (95% CI)** | **p-value** | **OR (95% CI)** | **p-value** |
| **Socio-demographic and medical characteristics at illness onset** | Age | 1.04 (1.01-1.07) | <0.01 | 1.02 (0.98-1.06) | 0.271 |
|  | Sex |  | 0.27 |  | *<0.01* |
|  | Male | Ref. |  | *Ref.* |  |
|  | Female | 1.59 (0.70-3.65) |  | *6.57 (1.85-23.34)* |  |
|  | BMI group |  | 0.98 |  | NA |
|  | Normal weight | Ref. |  | NA |  |
|  | Overweight | 0.74 (0.29-1.86) |  | NA |  |
|  | Obese | 1.08 (0.36-3.2) |  | NA |  |
|  | Number of high-risk COVID-19 comorbidities at illness onset* |  | *<0.001* |  | *<0.001* |
|  | 0 | *Ref.* |  | *Ref.* |  |
|  | 1 | *2.11 (0.74-6.03)* |  | *2.19 (0.60-7.96)* |  |
|  | 2 | *2.11 (0.56-7.95)* |  | *2.28 (0.48-10.87)* |  |
|  | 3+ | NA |  | NA |  |
|  | Presence of asthma & COPD | 0.18 (0.02-1.46) | 0.11 | NA | NA |
|  | Presence of other pulmonary comorbidities | 5.56 (0.88-35.20) | 0.07 | NA | NA |
|  | Smoking |  | 0.72 |  | NA |
|  | Non-smoker | Ref. |  | NA |  |
|  | Ex-smoker | 1.2 (0.51-2.82) |  | NA |  |
|  | Current smoker | 0.43 (0.05-3.72) |  | NA |  |
| **Clinical severity** | Clinical severity*** |  | *<0.01* |  | *0.02* |
|  | Mild | *Ref.* |  | *Ref.* |  |
|  | Moderate | *2.29 (0.60-8.70)* |  | *2.64 (0.60-11.66)* |  |
|  | Severe/critical | *7.78 (1.91-31.59)* |  | *7.17 (1.44-35.71)* |  |
|  | **Determinant** | **Univariable analysis** | | **Multivariable analysis** | |
|  |  | **OR (95% CI)** | **p-value** | **OR (95% CI)** | **p-value** |
| **Clinical features of COVID-19** | Presence of dyspnoea during PFT | *3.11 (1.24-7.80)* | *0.02* | NA | NA |
|  | Presence of fibrosis on CT during follow-up | 3.38 (0.70-16.17) | 0.13 | NA | NA |

* COVID-related comorbidities are based on WHO Clinical Management Guidelines [1] and include: cardiovascular disease (including hypertension), chronic pulmonary disease (excluding asthma), renal disease, liver disease, cancer, immunosuppression (excluding HIV, including previous organ transplantation), previous psychiatric illness and dementia. However, number of high-risk COVID-19 comorbidities at illness onset is not included in the multivariable analysis due to low participants with more than 3 comorbidities in one of the groups.

# **References**

1. World Health Organization. Clinical management of COVID-19: interim guidance, 27 May 2020. World Health Organization; 2020.
