# Supplementary figures and images for: One-fourth of COVID-19 patients have an impaired pulmonary function after 12 months of disease onset
Source: PLoS One. 2023 Sep 11;18(9):e0290893. doi: 10.1371/journal.pone.0290893 (PMC10495003; doi:10.1371/journal.pone.0290893)

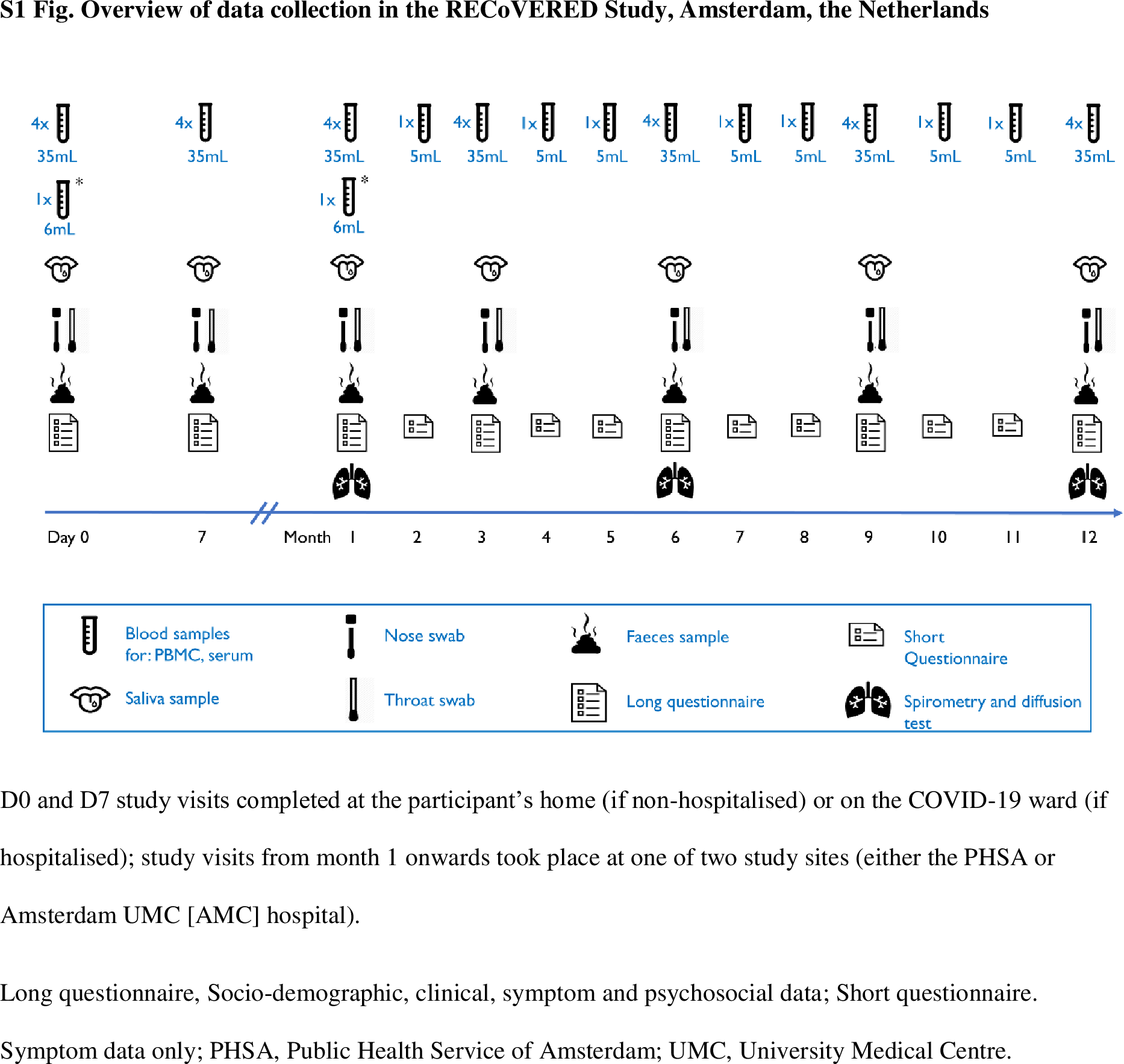

Supplement: S1 Fig — (TIF) [file pone.0290893.s004.tif]

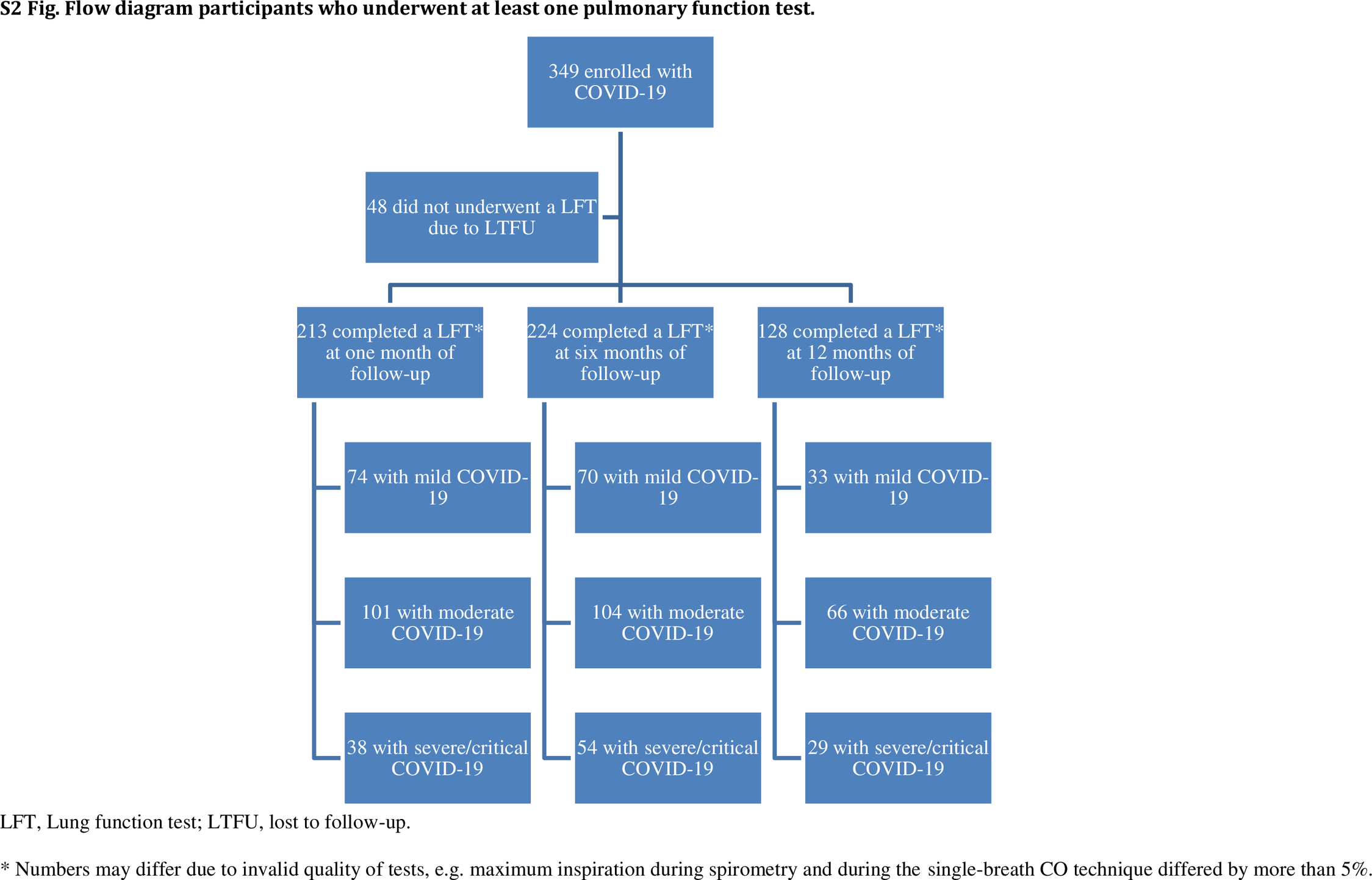

Supplement: S2 Fig — (TIF) [file pone.0290893.s005.tif]
